# Identification of novel compound heterozygous variants in isovaleric acidemia with hyperammonemia: Implications for continuous renal replacement therapy management

**DOI:** 10.1016/j.gendis.2026.102053

**Published:** 2026-01-24

**Authors:** Huan Huang, Ye Zhang, Limin Guo, Zijing Hu, Xiaolin Miao, Chao Lu

**Affiliations:** Department of Pediatrics, The First Affiliated Hospital of Nanjing Medical University, Nanjing, Jiangsu 210029, China

Isovaleric acidemia (IVA), a rare autosomal recessive disorder caused by isovaleryl-coenzyme A dehydrogenase (IVD, NM_002225.5) variants, leads to toxic isovaleric acid accumulation and hyperammonemia, causing multi-organ damage, primarily neurological impairment.^1^ IVA presentations include asymptomatic, late-onset chronic intermittent, or severe early-onset acute neonatal forms.^2^ Acute neonatal IVA has high mortality, often within the first two weeks. Hyperammonemia survivors due to inherited metabolic diseases face a high risk of severe neurological sequelae.^3^ Early diagnosis and intervention are pivotal for mitigating metabolic crises, reducing mortality, and improving neurodevelopmental outcomes. This study reports a Chinese family with neonatal IVA and hyperammonemia linked to a novel compound heterozygous *IVD* variant (c.149G>C and c.370C>T). Combining medication and continuous renal replacement therapy (CRRT) effectively normalized blood ammonia and achieved favorable prognosis. This identifies novel pathogenic *IVD* variants, expanding the known variant spectrum of IVA, and underscores CRRT's role in acute hyperammonemia management.

Blood acylcarnitines (C5, C5/C2) and urinary organic acids (isovaleric acid, 3-hydroxyisovaleric) were quantified using liquid chromatography-tandem mass spectrometry (LC-MS/MS) and gas chromatography-mass spectrometry (GC/MS), respectively.^3^ Whole-exome sequencing (WES) of peripheral blood DNA achieved high coverage (average depth ≥ 180X, > 95% > 20X). Identified *IVD* variants (c.149G>C, c.370C>T) were analyzed bioinformatically, classified per ACMG guidelines, modeled using SWISS-MODEL, and confirmed by Sanger sequencing. During acute hyperammonemia, continuous venovenous hemodialysis (CRRT/CVVHD) was administered until ammonia normalized (≤ 100 μmol/L) and neurological status improved.

A full-term male infant (3.5 kg), cesarean-delivered to non-consanguineous parents, initially thrived on breast milk but developed poor feeding on day 8. Admitted on day 9 with lethargy, diminished reflexes, irregular breathing, jaundice, and “sweaty feet” odor, he received initial treatments (antibiotics, phototherapy, fluids/calcium). His condition deteriorated on day 10 with convulsions, hypotonia, and absent primitive reflexes; phenobarbital and meropenem were administered. Diagnostic investigations revealed cerebellar hemorrhagic lesions on MRI, elevated cerebrospinal fluid protein (1971 mg/L), and cardiac abnormalities (sinus bradycardia, ST-T/QT changes). On day 11, he developed bradycardia, hypoxemia requiring mechanical ventilation, and hyperammonemia (589 μmol/L), prompting transfer for suspected inherited metabolic disorder.

Upon admission with hyperammonemia (593 μmol/L) and convulsions, the child received levocarnitine (200 mg/kg/day), glycine (400 mg/kg/day), arginine, high-dose B vitamins, protein restriction, and immediate CRRT. CRRT rapidly reduced ammonia to 148 μmol/L (12 h) and 25 μmol/L by (60 h), optimizing fluid balance. Mental status and breathing improved significantly with increased activity. Concurrent supportive therapies (liver protection, nutrition) stabilized electrolytes and creatinine. By discharge (day 34), ammonia was normalized (29 μmol/L; Fig. 1A), appetite restored and neurological status was normal. Serial biomarker monitoring (days 2–133) demonstrated progressive normalization of C5, C5/C2, and urinary IVG (Fig. 1B–D), confirming therapeutic efficacy.

**Figure 1:**
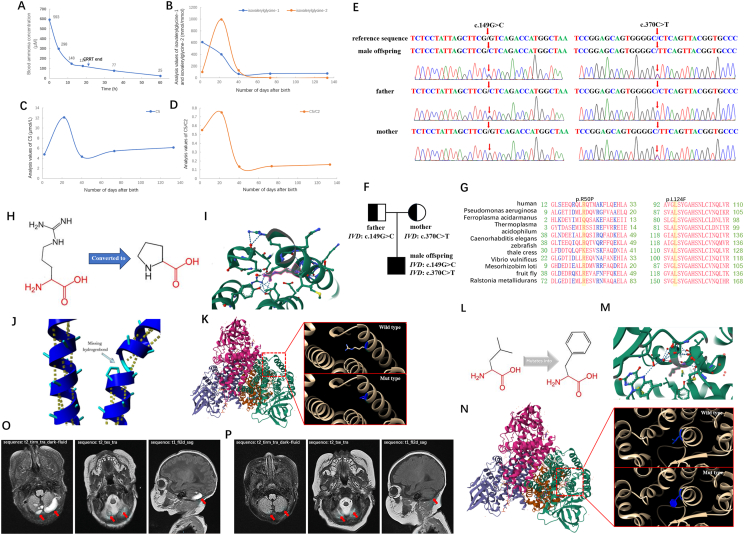
Integrated diagnostics and CRRT treatment of isovaleric acidemia (IVA). **(A)** Monitoring of blood ammonia concentration over time. **(B**–**D)** Monitoring results of blood C5 and C5/C2 levels and urinary isovalerylglycine levels. Isovalerylglycine-1 and Isovalerylglycine-2 denote isovalerylglycine with one and two derivatizing groups, respectively. Measurements were taken on different days. **(E)** Sanger sequencing chromatograms of the proband and family members around the site in the *IVD* gene. The arrow indicates the site of variation. **(F)** Pedigree of the family. Squares and circles denote male and female individuals, respectively. **(G)** Multiple sequence alignment of the isovaleryl-coenzyme A dehydrogenase (IVD) amino acid sequences, generated from the NCBI conserved domain database. Amino acids altered by the variants p.R50P and p.L124F are highlighted in yellow. **(H)** Schematic structures of the wild-type (left) and the mutant p.R50P (right) amino acid at position 50 of the IVD protein. The common protein backbone is colored red; the unique side chain is colored black. **(I)** Interaction diagram of wild-type residues Arg50 with surrounding residues in three-dimensional space. The red line outlines the wild-type residue. **(J)** Schematic of the effects of wild-type (Arg50) and mutant (Pro50) residues on hydrogen bonding and structural stability within an α-helix. **(K)** Predicted protein diagram and 3D structure model of the wild-type and mutant (p.R50P) IVD protein, generated using HOPE and Chimera software. **(L)** Schematic structure of the wild-type (left) and mutant p.L124F (right) amino acids at position 124 of the IVD protein. The common protein backbone is colored red; the unique side chain is colored black. **(M)** Interaction diagram of the wild-type residue Leu124 with surrounding residues in three-dimensional space. The red line outlines the wild-type residue. **(N)** Predicted protein diagram and 3D structural model of the wild-type and mutant (p.L124F) IVD protein, generated using HOPE and Chimera software. **(O)** Representative image of a plain cerebral MRI scan in the child before hospitalization. **(P)** Representative image of a plain cerebral MRI scan in the child after discharge.

Heel blood LC-MS at 48 h postpartum revealed elevated isovalerylcarnitine (C5: 4.75 μmol/L, reference: 0.04–0.4 μmol/L) and C5/C2 ratio (0.55, normal: 0–0.03), while urine GC/MS showed markedly increased isovalerylglycine (IVG-1: 609.16 mmol/mmol, IVG-2: 100.21 mmol/mmol), confirming IVA. While an acute-phase C5 elevations is characteristic, combined C5/C2 ratio and urinary IVG analysis is essential for differential diagnosis (*e.g.*, from short-chain acyl-CoA dehydrogenase deficiency) and to address isomer/antibiotics interference.^4^ The reports were received on day 15 post-birth (the fourth day of admission) and a targeted treatment was initiated. WES identified compound heterozygous *IVD* variants—c.149G>C (p.R50P) and c.370C>T (p.L124F)—in the proband, inherited from heterozygous parents (father: c.149G>C; mother: c.370C>T) (Table S1) and validated by Sanger sequencing (Fig. 1E).

Pre-hospitalization MRI revealed punctate hemorrhagic lesions in bilateral cerebellar hemispheres. Day 21 follow-up demonstrated bilateral posterior fossa subdural hemorrhages (left predominant) and multifocal cerebellar/vermis bleeding, with age-appropriate myelination (Fig. 1O). Repeat MRI at 2 months post-discharge showed partial hemorrhages resolution, residual cerebellar hemosiderin, and mild bilateral frontal hypoplasia (Fig. 1P). The observed hemorrhagic pathology was primarily confined to the cerebellum, vermis, and posterior fossa subdural spaces. While larger subdural collections exerted mass effect on the cerebellum, they did not result in significant distortion or signal changes within the brain stem parenchyma itself. Long-term management included leucine-restricted diet (1.5 g/kg/d natural protein + leucine-free formula), levocarnitine, and glycine. Serial anthropometric monitoring (length, weight, head circumference) at 1–6 months post-discharge confirmed normal development. A 2-month developmental test revealed age-appropriate social and cognitive milestones (total score: 90/100). To date, this regimen has successfully prevented metabolic decompensation, with sustained biochemical stability and no recurrent crises.

The pedigree is shown in Figure 1F. Cross-species alignment revealed evolutionary conservation at both variant sites (Fig. 1G). The p.R50P variant leads to a proline substitution at position 50 of the IVD protein (Fig. 1H). This variant (Fig. 1H) disrupts hydrogen bonding and ionic interactions (salt bridge with D37/E114/E115), causing misfolding (Fig. 1I–K). The website “HOPE” (https://www3.cmbi.umcn.nl/hope) and the software of Chimera were used to model the 3D conformations of wild-type and mutant IVD proteins (Fig. 1K). This variant disrupted conserved α-helix domains for protein folding (predicted pathogenic by CADD_phred, MutationTaster, MCAP, REVEL, LRT, and SIFT software, Table S2), and was classified as “pathogenic” in ClinVar (ACMG: PS3+PM1+PM2+PM5+PP3+PP5). Functional assessment showed reduced isovaleryl-CoA dehydrogenase protein levels and activity in the c.149G>C (p.R50P) variant.^5^ The novel p.L124F converts leucine to phenylalanine at position 124 (Fig. 1L), located in a mutational hotspot. This variant is absent from population databases (gnomAD/HGMD/ClinVar). The bulkier phenylalanine introduced steric clashes, perturbing ligand-binding interactions (Fig. 1M and N). Although initially classified as “variant of uncertain significance” using the WinterVar database (http://wintervar.wglab.org), it was upgraded to “likely pathogenic” (ACMG: PM1+PM2+PM3+PM5+PP3) due to trans-configuration with p.R50P. Both variants are located in critical α-helical domains (R50P in the N-terminus; the second in the conserved C-terminal domain, residues 356–403) and are predicted to disrupt key structural or oligomerization motifs.^1^ The proband exhibited classic IVA manifestations, including a “sweaty feet” odor (elevated unconjugated isovaleric acid in sweat) and hyperammonemia secondary to metabolic acidosis, consistent with pathogenic IVD dysfunction.

In summary, the integration of MS/MS and genetic testing has significantly enhanced IVA early diagnosis and timely intervention. This study underscores the clinical utility of integrated diagnostics and CRRT in IVA management while expanding the *IVD* variant spectrum. For neonates with hyperammonemia (> 200 μmol/L) accompanied by neurological symptoms (*e.g.*, seizures, lethargy) or refractory acidosis, current international guidelines—such as those by Häberle et al (PMID 30982989)—recommend the prompt use of ammonia-lowering agents. Carglumic acid is a first-line hyperammonemia treatment. When carglumic acid is unavailable, ammonia scavengers such as sodium benzoate serve as a critical alternative by conjugating with glycine to form excretable hippurate, thereby aiding in ammonia detoxification. Our experience supports the broader utility of CRRT in managing neonatal metabolic emergencies, especially in settings where specific antidotes are inaccessible. Prompt initiation of CRRT is advised to rapidly reduce ammonia levels, stabilize cerebral perfusion, correct metabolic disturbances, and reduce mortality. Although CRRT does not represent a cure for inherited metabolic disorders, it efficiently clears toxic metabolites (*e.g.*, isovaleric acid, ammonia, and their conjugates) during acute crises with a favorable safety profile.^3^ Its role is particularly important in settings where first-line therapies are unavailable or ineffective. Additional acute-phase interventions include high-dose glucose infusion, with insulin added as needed, to promote an anabolic state and mitigate endogenous protein catabolism, high-dose levocarnitine (100–300 mg/kg/day), glycine supplementation (150–300 mg/kg/day) to facilitate detoxification and excretion of isovaleryl-CoA, aggressive caloric support, and strict temporary protein restriction to limit leucine intake.^1^ These measures collectively aimed to halt toxin production, enhance elimination, and restore metabolic stability. Given autosomal recessive inheritance (25% recurrence risk), preimplantation/prenatal testing is advised. This case highlights the importance of early genetic screening, precision therapy, and multidisciplinary collaboration in neonatal metabolic emergencies. While CRRT is not a novel intervention, its timely application—as part of a comprehensive guideline-based approach—can be lifesaving in critical settings. These findings reinforce the need to strengthen availability and training for advanced supportive therapies like CRRT within regional perinatal care networks.

## CRediT authorship contribution statement

**Huan Huang:** Writing – original draft, Funding acquisition, Formal analysis, Data curation. **Ye Zhang:** Methodology, Investigation. **Limin Guo:** Formal analysis, Data curation. **Zijing Hu:** Visualization, Investigation. **Xiaolin Miao:** Writing – review & editing, Methodology. **Chao Lu:** Supervision, Project administration, Conceptualization.

## Ethics declaration

The studies were approved by the institutional review committee of the First Affiliated Hospital of Nanjing Medical University (No. 2021-NT-70). Written informed consent was obtained from the participants.

## Data availability

All data are available from the corresponding authors upon reasonable request.

## Funding

This work was supported by the third session of the Key Provincial Talents of Maternal and Child Health Program in Jiangsu Province (China), “333 high class Talented Man Project” (No. 2022-3-25-009) and the National Key Clinical Specialty Construction Project of China (Neonatology).

## Conflict of interests

The authors declare that they have no conflict of interests.
