# Supplementary material for: Identification of novel compound heterozygous variants in isovaleric acidemia with hyperammonemia: Implications for continuous renal replacement therapy management
Source: Genes Dis. 2026 Jan 24;13(6):102053. doi: 10.1016/j.gendis.2026.102053 (PMC13380702; doi:10.1016/j.gendis.2026.102053)
Supplement: Multimedia component 1 [file mmc1.docx]

Supplementary Materials for

**Identification of Novel Compound Heterozygous Variants in Isovaleric Acidemia with Hyperammonemia: Implications for Continuous Renal Replacement Therapy Management**

Huan Huang^*^, Ye Zhang, Limin Guo, Zijing Hu, Xiaolin Miao^*^, Chao Lu^*^

Department of Pediatrics, The First Affiliated Hospital of Nanjing Medical University, Nanjing, Jiangsu Province, China.

^*^Corresponding authors.

E-mail addresses: [huanghuan@njmu.edu.cn](mailto:huanghuan@njmu.edu.cn) (H. Huang), [miaoxiaolin@njmu.edu.cn](mailto:miaoxiaolin@njmu.edu.cn) (X. Miao), [luchaodoctor@163.com](mailto:luchaodoctor@163.com) (C. Lu)

**This file includes:**

Materials and Methods

Table S1 to S2

**Materials and methods**

**Study subjects**

The patient was the first child of a nonconsanguineous couple, delivered via caesarean at full term. The boy weighed 3.5 kg at birth and initially did well, being fed on breast milk for the first seven days. His parents noted that he had developed poor feeding on day 8. He was admitted to a local hospital on day 9, presenting with lethargy, poor reflex, irregular breathing, jaundice, and a characteristic “sweaty feet” odor. Cephalosporin oxime was administered as an anti-infection treatment, blue light was used for jaundice reduction, and calcium and fluid replacement therapies were administered. On day 10, the child experienced convulsions that lasted for approximately ten seconds each time, with lethargy, poor reflexes, low muscle tone in the limbs, and incomplete elicitation of the original reaction. Laboratory findings revealed severe thrombocytopenia (platelet count 69 × 10⁹/L) prompting the administration of an apheresis platelet transfusion (60 mL) to reduce the risk of life-threatening spontaneous hemorrhage. Coagulation studies indicated a profound coagulopathy, with prothrombin time >170 sec, activated partial thromboplastin time >170 sec, thrombin time >160 sec, fibrinogen level of 1.67 g/L, and D-dimer level of 1.09 mg/L. Fresh frozen plasma (60 mL) was promptly administered to address the clotting factor deficiencies. Fasting and fluid replacement were administered. Phenobarbital was used for seizure control, and meropenem for infection treatment. Head magnetic resonance imaging (MRI) showed scattered punctate bleeding lesions in both cerebellar hemispheres. Routine examination of the cerebrospinal fluid after lumbar puncture showed positive results in the Pan's test, and biochemical analysis indicated a total protein concentration of 1971 mg/L. Electrocardiography revealed sinus bradycardia, ST-T changes, and a prolonged QT interval. Oxygen therapy was administered, along with creatine phosphate sodium to nourish the myocardium, and dobutamine to enhance myocardial contractility. On day 11, the child experienced a decrease in heart rhythm and blood oxygen levels and was treated with invasive mechanical ventilation via a tracheal cannula. Blood ammonia was 589 μmol/L. Considering the possibility of inherited metabolic disorders, the patient was transferred to a hospital with a higher authority for further treatment.

**LC-MS/MS and GC/MS**

The levels of C5 and C2 in the blood of the patient were detected through LC-MS/MS using an API 3200 analyzer (Applied Biosystems, Foster City, Calif., USA), and the C5 and C5/C2 ratios were calculated. The samples were collected using the dry blood filter paper method (10). Urinary isovaleric acid and 3-hydroxyisovaleric acid were analyzed through GC/MS. A gas chromatograph was obtained from the Shimadzu Corporation, Japan (GCMS-QP2010 model) (11).

**WES and bioinformatic analysis**

The QIAamp DNA Mini Kit (Qiagen, Dusseldorf, Germany) was used to extract genomic DNA from peripheral blood lymphocytes of the patient and his parents. The DNA concentration was measured using a NanoDrop instrument and diluted to a concentration of 30 ng/μL for later use. The target region capture method was used to amplify the exons and flanking intronic regions, and a next-generation sequencing platform (Genome Analyzer IIx; Illumina, San Diego, Calif., USA) was used for sequencing. The average sequencing depth of the target area was ≥ 180X, and the proportion of sites with an average depth of > 20X in the target area was > 95%. Gene names recorded in the HUGO Gene Nomenclature Committee (HGNC) database were used, and the Human Genome Variation Society (HGVS) variant nomenclature (updated in 2016) was used to describe sequence variants. For mutation analysis and interpretation, we used pathogenic mutation databases such as ClinVar, HGMD, DECIPHER, ISCA, and NCBI; normal population databases such as gnomAD, ExACBrowser, and DGV; the Mendelian genetic disease database OMIM; and protein function prediction software MutationTaster, CADD_phred, LRT, SIFT, and REVEL. Gene variations within the detection range were screened and graded according to the American Society for Medical Genetics (ACMG) variation classification guidelines and supplementary guidelines. Mutation pathogenicity related or possibly related to clinical manifestations and the corresponding test results were rated (12). The online tool SWISS-MODEL (<https://swissmodel.expasy.org/>) was used to analyze the impact of single-base mutations on protein structure.

**Sanger sequencing**

The *IVD* gene variants (c.149G>C and c.370C>T) identified through WES were confirmed by Sanger sequencing. Primer pairs targeting each variant were designed with Primer Premier 6 software. For the c.149G>C locus, a 241-bp fragment was amplified using a forward primer (5’-ATC ATC TCT GAG GAG GCA TA-3’) and a reverse primer (5’-CAC TCG CAG GTT CTT GAA-3’). Similarly, a 343-bp fragment encompassing the c.370C>T site was amplified with a forward primer (5’-GCT GGT GAT GGA GGA GAT-3’) and a reverse primer (5’-GTC TGT CTG TTG TTC AAT GC-3’). PCR amplification was performed in 50 µL reactions containing 1× buffer, 1.5 mM MgCl2, 0.2 mM of each dNTP, 0.4 µM of each primer, 2 U of Promega Taq DNA polymerase, and genomic DNA template, using a PTC-225 Peltier Thermal Cycler (MJ Research, Watertown, MA, USA). Thermocycling parameters included initial denaturation at 94°C for 5min, followed by 35 cycles of 94°C for 30s, 55°C for 30s and 72°C for 25 s, with a final extension at 72°C for 7 min. After amplification, PCR products from the proband and parental samples were resolved on 2% agarose gels and subjected to Sanger sequencing. Sequence data were aligned against the *IVD* reference sequence (NG_011986.2) from the NCBI database (https://www.ncbi.nlm.nih.gov/) for variant confirmation.

**CRRT**

During the patient’s acute phase, general supportive care—including respiratory and circulatory support, correction of internal environment imbalance, and symptomatic treatment—was provided. Prior to the diagnosis, 10% glucose was administered for energy supplementation, along with arginine (100 mg/kg/day) to regulate amino acid metabolism, phenobarbital sodium for seizure control, and meropenem for anti-infective therapy. CRRT was initiated for the management of severe hyperammonemia, involving hemodialysis via a 6.5F double-lumen dialysis catheter in the right internal jugular vein, a Plasauto EZ machine (Asahi Kasei Medical, Tokyo, Japan), and an AEF-03 blood filter. The filter was pre-rinsed with heparin (0.9% NaCl solution) and the circuit was primed with a red blood cell suspension and fresh frozen plasma, matching the extracorporeal circuit volume. Treatment parameters included continuous venovenous hemodialysis (CVVHD) with conventional heparin anticoagulation (loading dose: 50 U/h; initial maintenance dose: 10 IU/h, adjusted to target an activated clotting time of 160–200 s), blood flow rate of 60 mL/min, dialysate flow rate of 1,000 mL/h (no ultrafiltration), and a commercial blood filtration replacement base solution (Qingshan Likang Co., Ltd.; National Medical Products Administration Approval No. H20080452) containing anhydrous glucose (10.6 mmol/L), total calcium (1.6 mmol/L), sodium (113 mmol/L), chloride (118 mmol/L), and magnesium (0.797 mmol/L), supplemented with 5% sodium bicarbonate (Solution B) and 10% KCl adjusted based on serum potassium. Vital signs, blood gas analysis, complete blood count, coagulation profile, and electrolytes were monitored every six hours during CRRT, which was discontinued when blood ammonia levels fell to 100 μmol/L and neurological function improved. Following the diagnosis of IVA, the infant’s feeding regimen was dynamically adjusted to a specialized formula (full specialized formula or specialized-to-regular formula ratios of 2:1/3:1) combined with controlled breastfeeding frequency/volume and leucine intake restriction.

**Post discharge treatment**

After discharge, dietary control and medications were administered to reduce leucine metabolites. The medication and dietary dosage were as follows: after discharge (34th day after birth), the first dose administered was special milk: breast milk 3:1, glycine 120 mg/kg.d, and levocarnitine 150 mg/kg.d; from the 40th day, special milk: breast milk 2:1, glycine 120 mg/kg.d, levocarnitine 150 mg/kg.d; from the 68th day, special milk: breast milk 2:1, glycine 200 mg/kg.d, levocarnitine 200 mg/kg.d; from the 115th day, special milk: breast milk 3:1, glycine 166 mg/kg.d, levocarnitine 110 mg/kg.d. The child's height, weight, and head circumference were regularly monitored. About a month after discharge, head MRI was performed.

Table S1 Gene variation information of the child

| Gene | Chromosomal location | Variation | Transcript number | Exon | ACMG rating | Father | Mother | Child | Inheritance patten |
| --- | --- | --- | --- | --- | --- | --- | --- | --- | --- |
| *IVD* | chr15: 40699841 | c.149G>C (p.R50P) ^1-3, 5^ | NM_002225.5 | Exon2 | Likely Pathogenic | Heterozygous | wild | Heterozygous | AR |
|  | chr15: 40702910 | c.370C>T (p.L124F) ^4-5^ | NM_002225.5 | Exon4 | Variant of Uncertain Significance | wild | Heterozygous | Heterozygous |  |

Table S2 Conservation prediction of the two variants by CADD_phred, SIFT, MutationTaster, GERP++, REVEL, MCAP, LRT, ClinPred, and MetaSVM_score

| HGVS | CADD_phred | SIFT | MutationTaster | GERP++ | REVEL | MCAP | LRT | ClinPred | MetaSVM_score |
| --- | --- | --- | --- | --- | --- | --- | --- | --- | --- |
| c.149G>C | Damaging(32) | Damaging(0) | Disease causing (1) | Conserved (5.27) | D(0.957) | P(0.69030708) | D(0) | 0.99771082 | 0.918 |
| c.370C>T | Damaging(27.2) | Damaging(0.001) | Disease causing (1) | Conserved (5.17) | D(0.851) | P(0.28292384) | D(0) | 0.99498308 | 0.993 |

**References**

1. D'Annibale OM, Koppes EA, Alodaib AN, et al. Characterization of variants of uncertain significance in isovaleryl-CoA dehydrogenase identified through newborn screening: An approach for faster analysis. *Mol Genet Metab*. 2021;134(1-2): 29-36.

2. Lee YW, Lee DH, Vockley J, et al. Different spectrum of mutations of isovaleryl-CoA dehydrogenase (IVD) gene in Korean patients with isovaleric acidemia. *Mol Genet Metab*. 2007;92(0):71-77.

3. Che FY, Yang Y, Wang Z, et al. Isovaleric acidemia due to compound heterozygous variants of IVD gene in a case. *Zhonghua Yi Xue Yi Chuan Xue Za Zhi*. 2021;38(2):150-153.

4. Li YH, Shen M, Jin Y, et al. Eight novel mutations detected from eight Chinese patients with isovaleric acidemia. *Clin Chim Acta*. 2019;498: 116-121.

5. Liu XM, Liu XQ, Fan WX, et al. Analysis of the genotype-phenotype correlation in isovaleric acidaemia: A case report of long-term follow-up of a chinese patient and literature review. *Front Neurol*. 2022;13: 928334.
